# Wear Debris Characterization and Corresponding Biological Response: Artificial Hip and Knee Joints

**DOI:** 10.3390/ma7020980

**Published:** 2014-02-10

**Authors:** Md J. Nine, Dipankar Choudhury, Ay Ching Hee, Rajshree Mootanah, Noor Azuan Abu Osman

**Affiliations:** 1 Department of Biomedical Engineering, Faculty of Engineering, University of Malaya, Kuala Lumpur 50603, Malaysia; E-Mails: heeayching@gmail.com (A.C.H.); azuan@um.edu.my (N.A.A.O.); 2 Faculty of Mechanical Engineering, Brno University of Technology, Technická 2896/2, Brno 61669, Czech Republic; 3 Medical Engineering Research Group, Department of Engineering and the Built Environment, Faculty of Science and Technology, Anglia Ruskin University, Chelmsford, Essex CM1 1SQ, UK; E-Mail: rajshree.mootanah@anglia.ac.uk

**Keywords:** wear debris, isolation, morphology, biological response, nano-toxicity

## Abstract

Wear debris, of deferent sizes, shapes and quantities, generated in artificial hip and knees is largely confined to the bone and joint interface. This debris interacts with periprosthetic tissue and may cause aseptic loosening. The purpose of this review is to summarize and collate findings of the recent demonstrations on debris characterization and their biological response that influences the occurrence in implant migration. A systematic review of peer-reviewed literature is performed, based on inclusion and exclusion criteria addressing mainly debris isolation, characterization, and biologic responses. Results show that debris characterization largely depends on their appropriate and accurate isolation protocol. The particles are found to be non-uniform in size and non-homogeneously distributed into the periprosthetic tissues. In addition, the sizes, shapes, and volumes of the particles are influenced by the types of joints, bearing geometry, material combination, and lubricant. Phagocytosis of wear debris is size dependent; high doses of submicron-sized particles induce significant level of secretion of bone resorbing factors. However, articles on wear debris from engineered surfaces (patterned and coated) are lacking. The findings suggest considering debris morphology as an important parameter to evaluate joint simulator and newly developed implant materials.

## 1. Introduction

Advancements in medicines and medical interventions in the last 60–100 years have reduced the aging process and increased human life expectancy [1]. This demands longer lifetime body support from the major body-bearing joints. Consequently, the estimated numbers of total hip and knee replacements (THRs and TKRs) are projected to increase by 673% and 174%, respectively, by the year 2030 in the USA [2]. However, patients are often affected in the post-surgery period (10–20 years after) by end-stage joint diseases, such as osteoarthritis and inflammatory rheumatoid arthritis [3]. One of the key factors that shortens the life of joint implants and increases the number of revision surgeries is wear debris, which is primarily generated at the bearing interface [4]. Wear debris also results in mechanical instability of the joint, reduces joint mobility, increases pain with detrimental biologic responses, results in osteolysis, and, ultimately, causes component loosening and implant failure [3–7].

New materials (Cross-linked polyethylene, carbon-carbon composite, carbon fiber-reinforced (CFR), polycarbonate-urethane (PCU), cobalt-chromium-based alloy (CoCr), titanium-based alloy (Ti), and ceramic-ceramic composite) and engineered surface (hard coating, dimpled surface, rectangular-patterned surface) with different sliding combinations have been introduced in total joint replacements (TJRs) in the last couple of decades to mitigate the risk of osteolysis. These inventions and improved material combinations have the potential to reduce the wear rates of implanted joints [8]. However, the revision rate remains high. For example, the UK national joint registry reported 8309 revision procedures, from 2008 to 2010, in England alone [9]. Similarly, the projected total hip and total knee revisions are to grow by 137% and 601%, respectively, between 2005 and 2030 in the United States [2]. The correlation among bone loss, wear debris, and secreted mediators [10] suggest that the interaction between the tumor necrosis factor (TNF-α) and the receptor activator of nuclear factor kappa-B ligand (RANKL) promotes osteoclast activity, which is associated with wear debris. In addition, the characterization of such implant wear debris is significant in predicting wear rate and understanding the wear mechanism of implant bearings [11,12]. The role of debris in the progression of aseptic loosening can be understood by debris characterization. The sizes, shapes, and chemical compositions of wear particles have been found to influence the responses of periprosthetic cells followed by subsequent complications. Subsequently, such bio-reactivity of wear debris into artificial joints can lead to considerable bone loss.

This review discusses and collates recent findings on detailed morphology of the particles obtained from hip and knee joints either *in vivo* or *in vitro*. The overview illustrates hip and knee implant materials and their tribology, as well as the common particle isolation practices from periprosthetic tissues (*in vivo*) and simulated body fluid (*in vitro*). In addition, the size- and dose-dependent biologic responses of debris are analyzed to provide a comprehensive review of information relevant to prosthetic wear debris.

## 2. Search Strategy

**Figure 1.**
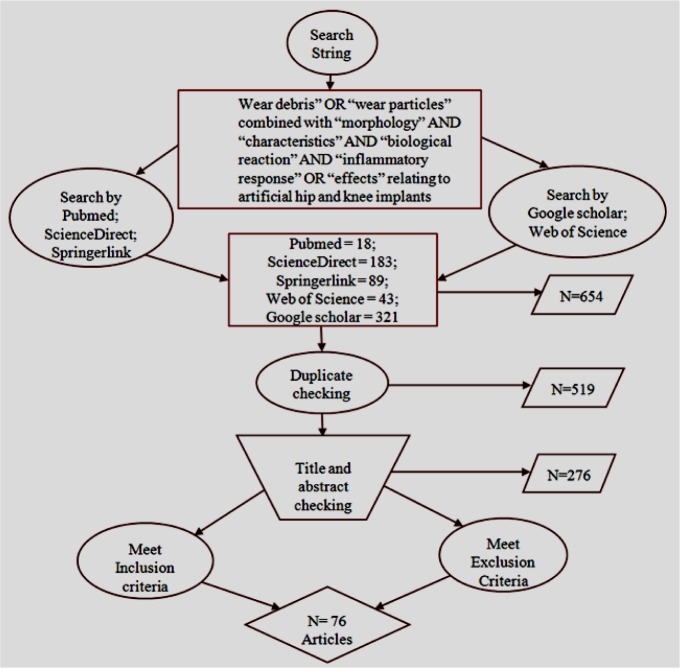
Flowchart illustrating the systematic search strategy of published peer-reviewed journals on wear-debris of hip and knee implants.

The available clinical and laboratory works on wear debris related to hip and knee prostheses and its biological reaction were considered in this systematic review. Articles, written in English and published in peer-reviewed journals, between January 2000 and December 2013, were considered eligible for this review. Databases, such as Pubmed, ScienceDirect, Springerlink, Web of Science, and Google Scholar, were searched, using the search string “wear debris” OR “wear particles” combined with “morphology” AND “characteristics” AND “biological reaction” AND “inflammatory response” OR “effects”, relating to artificial hip and knee implants under aseptic loosening. An extensive study was accomplished through advanced and individual search, which maximized the possibility of obtaining relevant articles. Individual search was conducted by following the additional bibliography of a specific author of an individual topic. The flowchart, shown in Figure 1, illustrates the analytical approach of the search strategy.

### Inclusion and Exclusion Criteria

This systematic review includes the following key points: (1) hip and knee implant materials and their debris formation mechanism; (2) debris from different hip and knee artificial joints; (3) particle isolation methods; (4) quantitative analysis of wear debris; (5) morphology of particles retrieved from hip and knee joints; (6) human periprosthetic cells and mediators and (7) *in vitro* inflammatory response to foreign particles.

Articles or the part of articles focused on any of the following criteria were considered beyond the scope of this review: (1) biological responses that are only limited to animal cells (murine/rats); (2) implant debris from shoulder, ankle, spinal joints; (3) prediction by numerical or computational analysis and (4) modeling of prosthetic joints and lubrication characteristics (except from those related to wear debris).

We also identified four partially-relevant review publications in this area, particularly on metal wear debris [13], particle isolation methods [14], and the biological response of orthopedic wear debris [15,16].

## 3. Hip and Knee Implant Materials and Their Tribology

Different types of polyethylene (ultra-high molecular weight polyethylene (UHMWPE), cross-linked polyethylene), metal (CrCo-based alloy, Ti-based alloy), and ceramic (Al_2_O_3_, ZrO_2_) biomaterials have been introduced in the last few decades to perform in hip and knee arthroplasty. Furthermore, design parameters of the joints, such as clearance and diameter, are also being investigated extensively and optimized. Surgical techniques have also been improved. Therefore, an orthopedic surgeon has a large number of options to select an appropriate implant for an osteoarthritic patient. However, based on material combinations, hip prostheses are classified as metal-on-metal (MoM), metal-on-ceramic (MoC), ceramic-on-ceramic (CoC), ceramic-on-metal (CoM), metal-on-polyethylene (MoP), and ceramic-on-polyethylene (CoP). Despite these improvements, the revision rate of artificial joints is still high, mainly due to excessive wear rate and the biological response of these wear debris.

Millions of wear particles in different sizes and shapes are generated annually from different artificial joint articulating surfaces and migrate to the periprosthetic tissues. In addition, abrasive wear of these joints can be promoted by third-body wear debris. The propensity for abrasive wear is found to be dependent on the relationship between the hardness of the third-body debris and the hardness of the bearing surfaces. Tribology of hip/knee joints is a complex mechanism, which involves a number of factors, including prosthesis material and geometrical properties, synovial fluid properties (various protein levels), patients’ lifestyles, and body weight. However, in this section we have focused on a simple tribology, based on the prosthesis material and geometrical properties.

### 3.1. Polyethylene

UHMWPE was first introduced as an implant material in the early 1960s by Sir John Charnley [17] when he developed the concept of low-friction arthroplasty. He was probably the first to identify polyethylene and cement debris in infected reconstructed joints [18]. Nevertheless, the initial success of UHMWPE as the cup material [19] has prevailed for 30 years, UHMWPE being the dominant orthopaedic material in total joint replacements (TJRs) [20]. Subsequently, macrophage and giant cells showed adverse response to the particles of polyethylene together with metal and acrylic cement debris [21]. Recently, the historic UHMWPE was replaced by the newer cross-linked polyethylene [22,23], which possesses superior mechanical properties with developed wear resistant characteristics [24,25]. Heiner *et al*. [26] investigated third-body scratches on both conventional UHMWPE and highly cross-linked polyethylene. They concluded that there was no significant difference between the two materials with respect to protection against severe scratching induced by large embedded third-body particles.

Two distinct wear mechanisms of UHMWPE, based on the scale of intimate asperity interactions, were reported by Wang *et al*. [27] that are operational in both total hip and total knee replacements. They revealed that the wear rate is strongly affected by the ultimate tensile strength and breaking elongation of the UHMWPE material. Particle detachment from bearing surfaces can be induced mechanically (repeated cyclic stress leads to fatigue) or chemically (changes microstructure in contacting surface) [28]. However, pitting and delamination were identified as the most common form of knee wear that can produce wear debris of a much larger scale [29,30].

UHMWPE with ceramic or metallic counter face causes stretching and reorientation on the crystalline and amorphous polymer phases. Often, a transfer of a thin film of UHMWPE on ceramic or metal counter face can result in lumpy shaped wear particles or granules, splinters, and flakes [31]. Adhesion between the liner and metal counter face generates fibrils on the surface that are later torn off by mechanical action, resulting in loose micro wear particles [32].

Surface roughness of implant surfaces were found to increase the propensity of wear and were associated with increased loosening rates [33]. Lately, *in vitro* and *in vivo* wear debris morphology was compared with associated wear mechanism for the same friction pair of UHMWPE and CoCr alloy [34]. Different shape and sizes of UHMWPE were defined as the consequences of different wear mechanisms. The larger particles are the outcome of adhesive wear, whereas the smaller particles are usually formed by the fragmentation of large wear debris or the exfoliation of surface micro-convex-bodies of friction pairs. Flat block shape and sheet/flake wear debris are found to be the results of adhesive and fatigue wear, respectively, whereas tearing wear debris (most irregular) is found to be the product of composite motion of friction pairs. Multi-directional motion imposes a higher wear rate of UHMWPE than reciprocating linear motion [35]. The crosslinking of UHMWPE reduces the degree of molecular orientation during sliding [36] and shows better wear resistance compared to conventional UHMWPE [37].

### 3.2. Metal

The first MoM hip prosthesis components were originally made of stainless steel [38], which was replaced by CoCr alloy to mitigate the excessive friction of the original sliding pair [39]. The second generation MoM THRs was introduced in the early 1990s to reduce polyethylene wear and to resist the rapid initiation of osteolysis [40]. Uses of CrCo alloy in MoM pair were shown to exhibit much less linear wear than MoP [41]. Even CoCr alloys were found to have less damage on UHMWPE than Ti-6Al-4V alloys [19,42] in MoP coupling. A study on MoP bearing with different metal couplings against polyethylene counterpart demonstrated different kinds of metal release rates. The linear wear rate of CoCr alloy was about 0.1 micron per year (10^6^ cycles), whereas the wear rate of 316L stainless steel and Ti-6Al-4V were in the order of 0.2 microns and 1 micron per year (10^6^ cycles), respectively [43].

Understanding the tribological mechanisms of metal components in TJRs is always important to improve the mechanical properties of sliding pairs. It is reported that the tribo-material formed in a nano-crystalline structure (having a thickness of less than 300 nm) when MoM hip joints articulate under ultra-mild sliding wear conditions incorporated with corrosion and fretting [44]. These tribo-materials have different chemical and mechanical properties than the bulk materials [45,46]. In addition, changes of surface wettability, oxidative wear of metal surfaces, micro-abrasion of metal surfaces from oxide film damage, and surface abrasion from third-body bone/PMMA debris affect wear rate and metal ion release from the metal surfaces in TJRs [43]. Recently, Wimmer *et al*. [47] reported that the nano-crystalline tribolayers of MoM components incorporate organic material stemming from the synovial fluid, termed as “mechanical mixing”. This mechanical mixing changes the bearing surface of the uppermost 50 to 200 nm from pure metallic to an organic composite material. It hinders direct metal contact (thus preventing adhesion) and limits wear. This finding of a mechanically mixed zone and organic constituents provides basic understanding of particle release from MoM arthroplasty.

In addition to material properties, geometry plays an important role in the tribology of MoM hip joints. For example, Leslie *et al*. [48] concluded that larger diameter MoM hip joints have lower wear rate compared to smaller diameter hip joints after a certain period of rubbing. Even, size of cobalt level was found to be higher in the smaller diameter hip joints after half-a-million cycles. Similarly, clearance was found be an influencing factor in MoM hip joints—a mean diametrical clearance of 94 μm had significantly lower friction and wear rate, followed by 53 and 150 μm diametrical clearances [49]. However, recent report showed that the number of complaints against the larger diameter hip joints is increasing in the UK [50], which indicates that the outcomes of *in vitro* tests do not always match those *in vivo*.

### 3.3. Ceramics

Orthopedic surgery employed ceramics for the first time in artificial hip and knee replacements in the early 1970s [51,52]. Recent trends indicate that CoC implants are likely to replace MoP because of their reduced risk of osteolysis, chemical inertness, and resistance to corrosion, low wear rates and non-allergic properties [53–55]. First generation ceramics implant used mechanically weaker Alumina (Al_2_O_3_) [56] and comparatively strengthened Zirconia (ZrO_2_) [57,58]; however, ceramic composites [59,60] are being studied intensively to improve their mechanical performances in TJRs by reducing their brittleness and slow crack growth [61] that led to joint failures [62] associated with variably-described sounds, namely, squeaking, pop, and click [63,64].

Grain pull-out is reported to damage the ceramic-bearing sliding surface which leads to higher surface roughness and increased friction in this area [65]. Macroscopic stripe wear is another common form of ceramic wear caused by edge loading. Grains are fractured out of the surface when the stripe wear appears to have resulted from the direct contact of the femoral head with the acetabular shell [62]. This contact is also the probable cause for the sudden onset of squeaking in the previously ‘silent’ hip articulation. Multiple smaller fragments are generated and, hence, the surface roughness increases, leading to a higher wear rate [66,67]. Grain pull-out occurred in ceramic prostheses, despite their better surface wettability properties than the conventional MoP bearing materials [68]. Squeaking of ceramic materials is found to influence wear mechanism of CoC hip joints. Currier *et al*. [69] found that the ceramic ball-in-socket bearing couple alone, without any metal devices incorporated, can be made to vibrate at an audible frequency when articulated. Consideration of the geometry of current generation CoC hip bearings led to a hypothesis of a rolling/sliding mechanism causing vibration and squeaking. In addition, a new mechanism of failure of a CoC THRs is reported by Bonnaig *et al*. [70], due to fretting corrosion and failure of the Morse taper. Failure of the Morse taper led to metal debris, which rubbed with the ceramic and caused dramatic third-body wear. The malfunction of the Morse taper, as reported in this case, represents a possible failure mechanism of a CoC THR.

### 3.4. Hard Coating and Textured Surface

Hard coating on bearing surfaces is another option to fabricate a mechanically superior and highly biocompatible surface for implanted bearing [71]. Diamond-like-carbon (DLC) is a coating material with good wear resistance and chemical inertness properties [72]; such ideal materials were proposed for protecting implants more than a decade ago [73]. In addition, the improved biocompatibility and reduced ion release with better wear-resistant properties of titanium and chromium nitride coatings [74–76], tantalum-based multilayer coating [77,78], carbon ion implantation (CII) coating [79], and amorphous diamond coatings [80], on conventional metal bearing have been investigated. Other surface engineering techniques are found to be effective to reduce friction and wear properties in local contact area of sliding pair—patterning concave dimples on polyethylene [81]; wavy, square grid and simple dimpling on metal and ceramics bearing surface [82], and modeling circular pattern [83] are found to be significantly effective to improve boundary lubrication and wear resistance of bearing surfaces.

The wear mechanism of such hard coatings have been studied and revealed different results. Sliding-induced heat accumulating on local contact areas of DLC can possibly cause a gradual destabilization of the carbon-hydrogen bond in the sp^3^ tetrahedral structure of DLC [84]. The movement of hydrogen atoms can thus trigger the transformation of the sp^3^ structure in to a graphite-like sp^2^ structure. Such graphitization of DLC is promoted by thermal and strain effects under higher load. The repeated cyclic wear then damages the secondary film formed on DLC [85]. Tribo-oxidation is discussed as another mechanism of such hard coatings under different tribological environment [86].

## 4. Wear Debris Isolation Protocol

**Figure 2.**
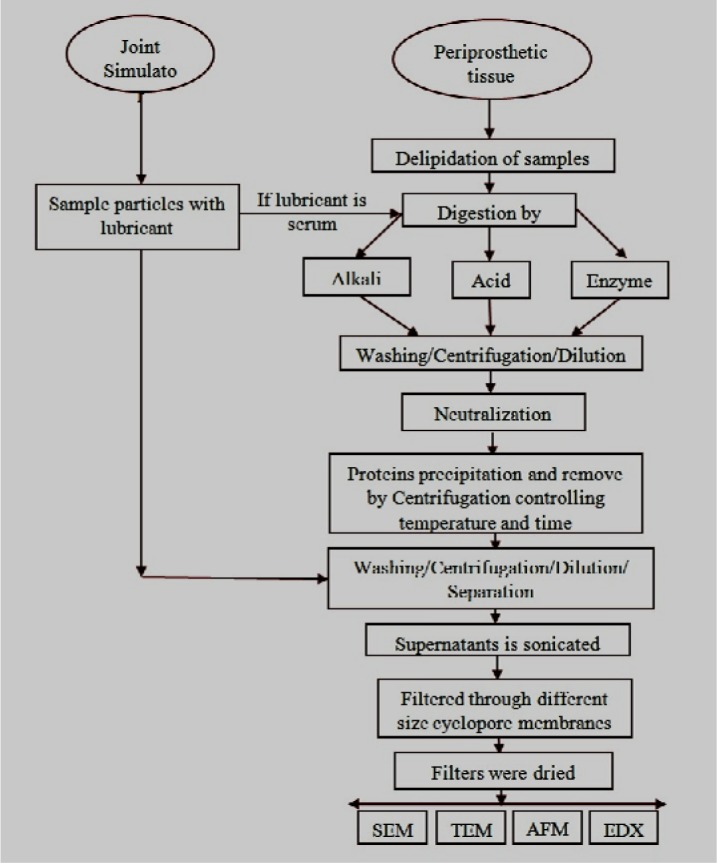
General method of Particle Isolation.

Generally, the particles are isolated from organic tissues (*in vivo*) and from simulated body fluids (*in vitro*) before characterization. Isolation protocols must be varied with the particle materials. Polyethylene, metal, and ceramics particles have different individual isolation protocols, as shown in Table 1. Nevertheless the reported common steps of particle isolation protocol are categorized into three different stages [87–105]: (Step 1) sample delipidation and tissue digestion; (Step 2) dilution, centrifugation, and protein separation and (Step 3) ultrasonication and debris separation. These steps are summarized from a regular chain of a continuous isolation process and are illustrated as a flowchart (Figure 2).

### Step 1. Sample Delipidation and Tissue Digestion

The collected and preserved (freeze-dried) biopsy samples were harvested into small segments and were washed with chloroform/methanol for lipid or lipid group removal. The extracted tissue samples were then dried. This pre-stage of tissue digestion (delipidation) is reported in several studies [91,92].

The digestion of tissues and sera free the particles from a sticky host. The chemical methods, namely, alkaline and acidic digestion, as well as enzymatic digestion, have been employed for the last 15 years to digest organic tissues and sera to isolate metal, ceramic, and polyethylene particles. The application of the different available digestion processes for different materials are shown in Table 1.

The suitable approach of debris isolation from periprosthetic tissue or simulated body fluids depends on the material and medium of the debris. In fact, all three approaches (alkaline, acidic, and enzymatic digestion) can be applied to ceramics (metal oxides or carbides), which are chemically inert. The acidic protocol remains popular [88,107] for isolating ceramic particles from periprosthetic tissues. However, the detrimental effects of aggressive alkaline solution on CrCo alloy particles were reported [93,94] as metals are prone to being ionized and oxidized. On the other hand, the enzymatic protocol allows the superior isolation and characterization of metal particles without affecting the shape and size of particles [92–94,98]. Comparisons of these three approaches were individually studied [89,91,100] and the relevant discrepancies were reported. Niedzwiecki *et al*. [89] reported that the enzyme method generated the least amount of hazardous waste compared to chemical (alkaline and acidic) protocols; thus, an optimized enzyme method was suggested as a practical standard for debris isolation and analysis.

Slouf *et al*. [91] found that the acid method was the most convenient, given the time needed for isolation, the cost of chemicals, and the final purity of the isolated particles. Baxter *et al.* [100] showed that 5 M NaOH, 5 M KOH, and 15.8 M HNO_3_ enabled the most complete digestion of human hip tissues and highlighted the enzymatic protocol for perfect digestion.

### Step 2. Dilution, Centrifugation, and Protein Separation

The sample was aspirated, heated, and then diluted by chloroform and methanol before centrifugation to separate the remaining contaminating proteins and lipids after digestion. In fact, the three digesting methods more or less applied centrifugation to separate the particles from the digested tissue solution [92–96]. Such centrifugation process enables separation of different particles, based on their density level. A method was developed to avoid centrifugation, based on the digestion of paraffin-embedded tissue samples, because of the possibility of morphological changes in the particles during centrifugation [101]. However, centrifugation speeds up to 105,000× g were later found to have no effect on the morphology and quantitative image analysis parameters, such as equivalent diameter, circularity, and elongation [102].

### Step 3. Ultrasonication and Particle Separation

Particles with excessive contamination were made agglomeration-free and were uniformly dispersed into the solution through ultrasonic action [88,92]. The dispersion solution was subjected to vacuum filtration [90,91] at different nanometer to micrometer pore sizes after the confirmation of quality. The use of different filtration sizes limited the particle sizes in the same cohort. The filter paper with particles and the solution with particles of limited sizes were then dried.

A few articles were identified on histological analysis of particle characterization, which does not require the isolation protocol. Solis-Arrieta *et al*. [108] determined the composition of the debris materials, using energy dispersive X-Ray analysis (EDX), following the conventional histological technique. Laser capture micro-dissection into periprosthetic tissue [103] and transmission electron microscopy (TEM) [104] were also employed to characterize the intercellular particles.

## 5. Debris Characterization

The filtered particles were prepared for morphological characterization and were subjected to instrumentation for image and data acquisition. The different types of morphological tools employed for particle characterization are summarized in Tables 2 and 3.

### 5.1. Debris Morphology Based on *in Vivo* and *in Vitro* Analysis

**Figure 3.**
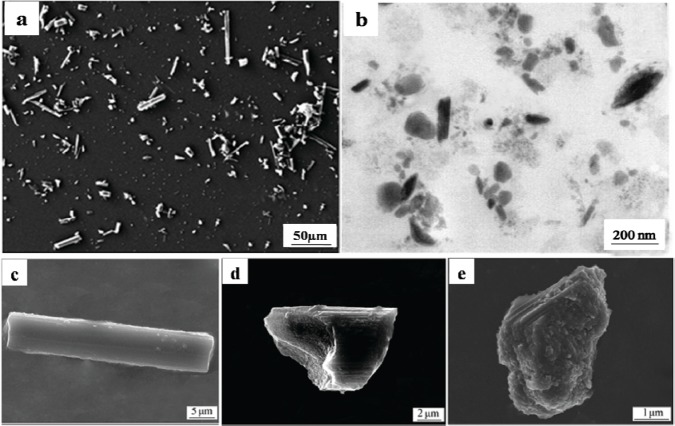

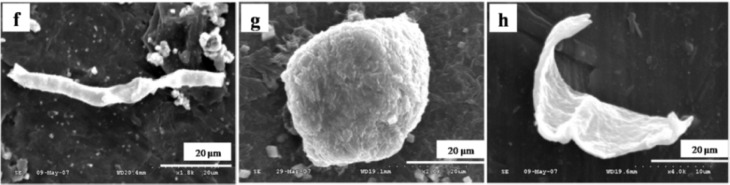
Typical morphologies of debris from joint simulator; (**a**) Carbon/Carbon composites [110]; and (**b**) CrCo alloy [111]; (**c**) Cylindrical (C/C composites) [110]; (**d**) Radial broken (C/C composites) [110]; (**e**) Blocky/Slice (C/C composites) [110]; (**f**) Fibril and Twig (UHMWPE) [34]; (**g**) Spherical (UHMWPE) [34] and (**h**) Sheet/flake type (UHMWPE) [34].

**Figure 4.**
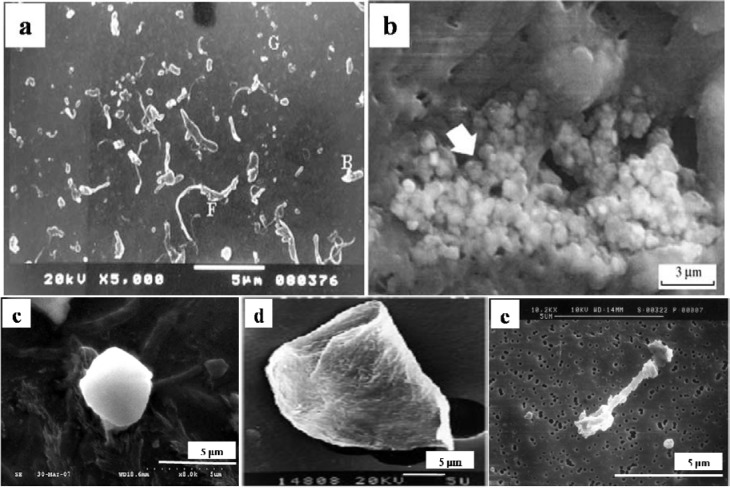
Typical morphologies of wear debris from periprosthetic tissue; (**a**) UHMWPE [90]; and (**b**) Alumina [103]; (**c**) Spherical (UHMWPE) [34]; (**d**) Sheet/Flake type (UlHMWPE) [112] and (**e**) Fibril (UHMWPE) [101].

The particles isolated from the simulators and periprosthetic tissues appeared to be predominantly submicron in size [109] and had both regular and irregular shapes, as shown in Figures 3 and 4. The sizes and shapes of these particles were found to vary between *in vivo* and *in vitro* analysis. Nevertheless, the evaluation of *in vitro* tribological studies is justified as they reproduce *in vivo* results.

However, Catelas *et al*. [111] concluded with partial uncertainty that CoCr particles retrieved from MoM joint simulator were very similar in composition, length and shape to the particles retrieved from MoM joint of patients. The common shapes of the particles retrieved from joint prosthetics were found spherical, flake, and fibril (Figure 4), whereas the joint simulator generated cylindrical, radial broken, block, fibril/twig, spherical sheet, and flake [34,110], as shown in Figure 3. Hongtao *et al*. [34] reported the *in vivo* and *in vitro* difference of particle sizes from UHMWPE and CoCr alloy friction pairs. They found that UHMWPE particles from joint simulator were larger in size (average diameter of 6.89 μm) than the particles isolated from the periprosthetic tissues (average diameter of 1.33 μm, which is about18% the size of the debris from the joint simulator). Buscher *et al*. [44] found that the majority of the CoCr wear particles *in vitro* were globular with a diameter <100 nm, whereas the mean diameter of the *in vivo* particles were <80 nm and had a minority of particles that were needle-shaped in both of the cases identified by a scanning electron microscope (SEM).

### 5.2. Debris Morphology Based on Bearing Types and Bearing Size

The differences in wear mechanisms and wear outcomes between hip and knee should be attributed to the difference in loading and sliding configurations with different degree of freedoms influencing debris morphology. Knee prostheses were found to produce larger UHMWPE particles with the mode of particle size in the 0.1–1.0 μm size range, compared to <0.1 μm size range for hip prostheses [96]; however, there was no significant difference in wear rate between these two joints. In addition, Benz *et al*. [104] reported that more than 75% of the UHMWPE particles retrieved from the hip joint had a length <0.5 μm, but only 43% of the UHMWPE particles from the knee joints were <0.5 μm in length. Similar results were found by Mabrey *et al*. [99] who reported that the particles from the hip joint had an equivalent circular diameter (ECD) of 0.694 ± 0.005 μm, which is relatively smaller than those retrieved from the knee joints (ECD of 1.190 ± 0.009 μm). Furthermore, the debris sizes were found to be influenced by the bearing type and bearing size. Some investigators suggested that mobile bearings were [109,113] advantageous over fixed bearings, based on their wear behavior and improved kinematics. However, no significant difference was found in wear rate and debris size between the mobile and fixed bearings of knee prostheses, using knee simulators [114,115]. Therefore, the previous suggestion was rejected. Leslie *et al*. [48] reported that debris size, wear rate, and ion levels were not influenced by bearing sizes. They conducted an *in vitro* investigation on 39- and 55-mm diameter MoM bearings. The investigation showed no significant differences in mean particle size (ranging from 8 nm to 108 nm and having round/globular shape) derived from both bearings. No needle-shaped particles were observed. The ion levels measured suggested both bearing sizes had similar initial wear rate; and the 55-mm diameter bearing reached steady state wear more rapidly than the bearing of 39 mm. However, a previous study on MoM bearings reported that 56-mm bearings produce reduced-sized particles compared with 28 mm bearings [116].

The aforementioned findings from the different studies show different results on debris morphology and wear behavior derived from different sizes and types of weight-bearing joints. However, it is accepted that debris characterization can be a parameter to optimize bearing sizes for different weight-bearing joints.

### 5.3. Debris Morphology Based on Bearing Materials

UHMWPE, metals, and ceramics were found to be the predominantly-studied materials for debris characterization (Tables 2 and 3). The cross-linking of UHMWPE definitely improved wear resistance [23,117], indicating successful material development. Therefore, highly cross-linked UHMWPE was found to produce >90% fewer wear particles in large size ranges and smaller-sized particles than the conventional UHMWPE [24]. However, a counter finding showed [118] no significant difference between cross-linked and non-cross-linked UHMWPE in the percentage number and percentage volume of particles in the size ranges tested in a multi-directional pin on a plate wear simulator.

Most of the studies on UHMWPE debris report material characterization of a large range of sizes (0.1 to 10 μm) [101,104,105,119–121] with irregular-shaped particles of higher aspect ratio [114]. Nano-sized (18.5–21.2 nm) UHMWPE wear debris were recently investigated *in vivo* for the first time by Lapcikova *et al*. [92]. These UHMWPE particles were found to have the most irregular shapes compared to those from MoC bearing surfaces, such as, fibril, flake, cylindrical, globular, twig, and, sometimes, spherical shapes, as summarized in Tables 2 and 3.

Metal particles retrieved from MoM implants were found to be smaller in size than polyethylene debris from MoP joints. The hip simulator for CoCr alloys with different carbon contents generated metal particles in the range of 25 nm to 36 nm [122]. A similar outcome was reported *in vivo* by Brown *et al*. [93], who indicated that most of the generated debris retrieved from hard-on-hard (MoM and CoM) hip prosthesis were less than 50 nm with round and irregular morphology. Wear debris with sizes ranging from 30 nm to 100 nm were also found *in vitro* [98,123] with mostly round to oval shapes and some needle shapes. Milosev and Remskar [106] also identified needle-shaped particles, ranging from 40 nm to 120 nm and containing both Co and Cr, isolated from the periprosthetic tissue of the MoM bearing. The globular particles reached 90 nm and contained high levels of Cr and no Co.

Wear debris concentrations from CoC hip joints *in vivo* were two to 22 times lower than those of MoP and CoP [107]. An earlier study, conducted by Mochida *et al*. [88], reported that no significant difference in the average size exists among the different types of particles retrieved from either CoC or CoP hip prostheses. The nanometer-sized ceramic wear particles in retrieved tissues were first reported to [103] range from 5 nm to 90 nm in size, measured by TEM. However, studies using SEMs, which have lower resolution than TEMs, revealed ceramic wear particle sizes ranging from 0.05nm to 3.2 mm. The presence of very small alumina wear debris (2 nm to 27.5 nm) was noticed during the micro-separation of the prosthesis components of the CoC joint [56].

**Figure 5.**
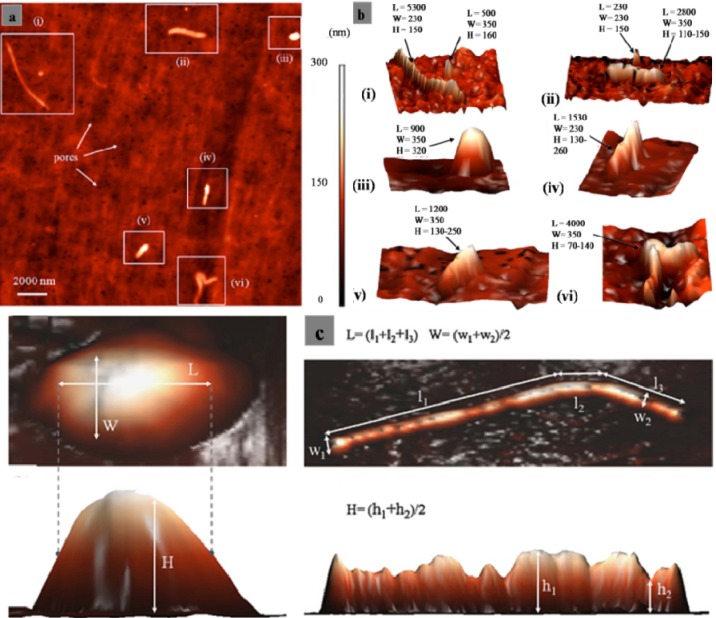
AFM morphology of UHMWPE wear debris [126]; (**a**) A two-dimensional projection of AFM data for debris particles of the 0.2–0.8 μm fraction precipitated on a filter. Six of the larger particles and three pores are indicated; (**b**) three-dimensional projections of AFM data for the six particles indicated in pane (dimensions are in nm); and (**c**) examples of length (L), width (W), and height (H) measurements on two representative UHMWPE particles.

The type of lubricants used in the joint simulator influenced the shape and size (length) of the debris. Serum produced smaller and thinner particles in size than the particles produced in water as lubricants for metal [122] and polyethylene [124] materials. Wear particle size was found to remain unchanged with changes of head-cup pair material, despite being considerably affected by wear rate. The change in the head materials in a hip joint simulator did not show any effect on debris size distribution [123]. The influence of head roughness on wear particles was evident and showed an increase in minimum particle size and surface roughness. Atomic force microscopy (AFM), along with SEM and TEM imaging techniques, added a new dimension in the debris characterization. A 3D size and shape characterization of UHMWPE wear debris was recently presented [125], although Scott *et al*. [109] previously introduced the AFM to improve the estimation of UHMWPE volumetric wear rate *in vitro*. A MiaoXAM2.5X-50X ultra-precision contourgraph was used to investigate the 3D morphology and thickness of the wear debris [34]. Gladkis *et al*. [126] subsequently showed the quantification of the size and shape of UHMWPE wear debris in all three spatial dimensions (Figure 5). The investigation clearly defined the length L, width W, and height H measurements. The approach was a sensible compromise between the practical considerations of the AFM technique and the correct determination of the particle dimensions.

### 5.4. Quantitative and Statistical Analysis of Wear Debris

Wear debris distribution was not homogeneous throughout the tissues because of clumping and clearing of the debris through drainage. The number of particles collected per unit of wet tissue was highly dependent on the biological variations of the tissue [112]. Therefore, randomizing the harvested tissue samples became a general practice before digestion. In addition debris morphology may largely be influenced by the lack of experimental precision because of different types of quantitative methods.

The common parameters in defining each particle employed by most of the researchers were ECD, roundness (R), form factor (FF), aspect ratio (AR), and elongation factor (E) [92,99,101,112,115,127]. Most of the studies used 2D SEMs and TEMs as the input to obtain quantitative statistics. The American Society for Testing and Materials **(**ASTM) F1877-98 [113] outlines that fractal dimension was sometimes accounted for the characterization of the morphology, number, size, and size distribution of the particles. Tipper *et al*. [112] determined the total number of particles, using a mean thickness value, the mean area of the particles, the density of the material, and the mass of the debris on the filter. SEM was later used to develop an automated quantification method (SEMq) with errors less than 10%, as verified with several sets of experiments [91]. The SEMq methods indicated that the distribution of UHWMPE particles around the total joint replacements was non-homogenous. Slouf *et al*. [128,129] later introduced infrared spectroscopy (IR) and LSC (Light Scattering with Calibration spheres) methods to determine the total volume of the UHMWPE and number of wear debris produced. The results showed good correlation with the radiographic appearance and indicated that extended tissue damage in a particular zone around the total joint that was proportional to the volume of the wear debris in that zone. Another extraction method of analysis is laser diffraction particle analysis [87], which has advantages in retaining the particles in the solution produced by the purification technique that avoids agglomeration and contamination [123]. Three-dimensional imaging approaches for particle quantification were reported in several studies [34,109,125,126]. Figure 5 outlines the 3D quantification of the particle measurement of different shapes and sizes described by Gladkis *et al*. [126].

From the debris morphology, data were extracted, organized, and interpreted to create a graphical presentation. Normal distribution was commonly reported, using the mean and standard deviation of length and width [105,111].Numerous studies represented the particle area, maximum dimension (length) [102,112], and volume distribution [118,123,129], using particle size and number.

Very recently, the impact of different methodologies was compared by Schröder *et al*. [130]. They concluded that particle characterization is a complex analytical method with a multiplicity of influencing factors. It becomes apparent that a comparison of results of wear particles among different research groups is challenging.

## 6. Biological Responses of Wear Debris

Debris with nano- to micro-sizes with different shapes [124,131] affected the secretion of different inflammatory mediators by the periprosthetic cells. Numerous studies and new findings are available to discuss [23,108,120,121,131–155] the reaction between the periprosthetic cells and prosthetic wear particles.

### 6.1. Cell, Mediators and Biologic Assay

Chronic inflammatory response, initiated by particulate debris at the implant-bone interface in a wide array of cell types, limited the longevity of joint reconstruction. These cells include macrophages, fibroblasts, giant cells, neutrophils, lymphocytes, and, most importantly, osteoclasts [132] studied *in vitro*.

Cells, cultured with particles of different materials [133,134] with different sizes [135–137], shapes, and doses [138], secreted different types of functional inflammatory mediators that acted locally at the site of cell damage and infection [136,139,140]. After activation by the wear particles, the phagocytes produced inflammatory mediators/secreted factors such as TNF-α, RANKL, IL-6, PGE_2_, and IL-1b, which are implicated in osteoclast activation and bone resorption [140,141]. The expression of bcl-2, bax, and caspase-3 was studied to understand the mechanisms that lead to apoptosis in macrophages. Bcl-2 is considered a death-regulating gene. Bax has a powerful death-promoting ability for cells. Caspase-3 is probably the most correlated with apoptosis among the different proteases [141,142]. Human osteoblast and fibroblast with metal alloy and ions were also studied [133,143–146] to investigate cytotoxicity and genotoxicity. The tests were related to cell viability, proliferation, alkaline phosphatase activity (APL), DNA damage, and chromosome aberrations. The formation of mineral nodules into cells was also identified [145]. Zymography analysis [141] was conducted to reveal the protein expression of cells affected by ions or debris.

### 6.2. Particle Size, Shape and Dose Dependent Cell Response

Phagocytosis of the particles was found to be correlated with changes in particle morphology. Cell proliferation, differentiation, and prostanoid production were affected by size, shape, and dose of wear particles. In addition, the chemical composition of particles from metal alloy and polyethyleneare found to affect alkaline phosphatase and PGE_2_ [133]. Previous studies have suggested that small polyethylene particles (less than 1 μm) can be more easily phagocytized than larger particles, and elongated particles may induce a stronger cellular reaction than round particles [5,6]. However, no statistically significant differences in (*in vitro*) biologic responses were noted between highly cross-linked and conventional polyethylene debris at low and intermediate doses. Only at the highest dose tested, highly cross-linked polyethylene was significantly more inflammatory than conventional polyethylene, based on relative TNF-α and vascular endothelial growth factor secretion levels [156]. *In vivo* analysis by Illgen *et al*. [157] also showed that cross-linking increases the inflammatory response to similar-sized conventional polyethylene debris. Polyethylene particles with mean sizes of 0.21, 0.49, 4.3, 7.2, and 88 μm were co-cultured with cells for 24 h prior to the assessment of the cell viability and production of the osteolytic mediators, such as IL-1b, IL-6, TNF-α, GM-CSF, and PGE_2_ [136]. Cell viability was unaffected by UHMWPE particle sizes. Only particle sizes between 0.21 and 0.49 μm produced significantly enhanced cytokine secretion.

**Figure 6.**
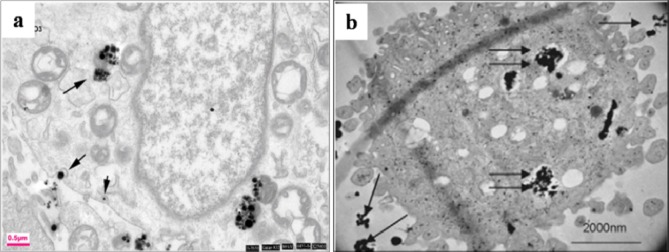
(**a**) TEM images of MG63 cells at 37 °C (incubation with Al_2_O_3_ NPs for 6 h), Arrows pointing to the process of internalization at the surface associated with actin rearrangement near the plasma membrane and extension into the extracellular space [150] and (**b**) SEM image of live primary human dermal fibroblasts exposed to CoCr alloy nanoparticles for 24 h outside and inside the cell [139].

**Figure 7.**
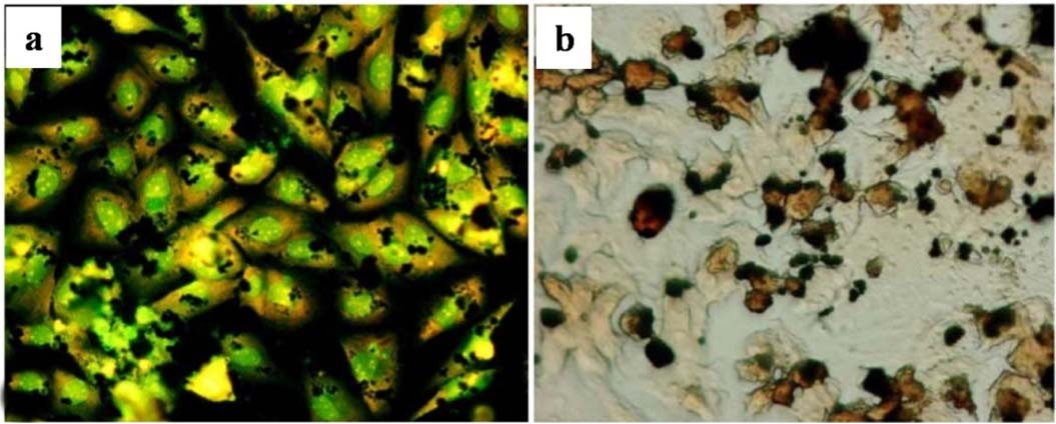
(**a**) Saos-2 cells challenged for 24 h with 0.5 mg/mL of FeAlCr alloys (avg. dia. 3.7 ± 0.4) and (**b**) Mineral formation after 21 days by Saos-2 cells added with 1 mg/mL of FeAlCr alloys [145].

The afore-mentioned study on particle characterization (Section 5) implied that MoC implants generate significantly smaller particles than MoP. Clinically-relevant CrCo alloy nanoparticles from MoM joints appeared to disintegrate within the cells faster than micro-particles. These nanoparticles (Figure 6) induced more DNA damage, aneuploidy, and cytotoxicity than micron-sized particles of an equivalent volumetric dose [139,158]. Metal nanoparticles from MoM hip joints [93,98,122,158] vastly increase the total surface area of the metal, which increases the propensity of releasing metal ions *in vivo*. The variation of cellular damage with different Cr (III) complexes ([Cr(en)_3_]^3+^) [146] inhibited cell proliferation of human dermal fibroblasts and causes intracellular damage through the formation of apoptotic bodies and chromatin condensation, all of which indicate cell death. The Co^2+^ and Cr^3+^ ions inhibited bcl-2 expression but stimulated bax and caspase-3 expression [141] at different periods of incubation with the macrophage. The release of soluble ions from CoCr particles was identified as the most likely cause for DNA damage within the first hour [144]. The overall level of DNA damage and structural aberrations caused by the CoCr alloy is approximately the same for both young and older cells. Older cells showed a greater loss of viability, induction of senescence, and a lower rate of mitosis and cell growth than young cells [143].The effect of the micro-sized particles of Ni-free Fe-based alloys resulted in mineralization into osteoblasts after 21 days (Figure 7), where the cells were overloaded with small particles in the cytoplasm [145]. Mineral nodules were observed all over the surface of the multi-layered cells, despite being fewer in number than the unexposed cells. The viability and proliferation of the osteoblast were found substantially unaffected by the presence of the particles of the FeAlCr alloys, which were phagocytized, based on size. The ion release rate from the aluminum and chromium particles in the culture medium increased with higher doses.

Nano-toxicity is now highly linked with osteolysis. Knee prostheses are thought to have lower osteolytic risks compared to the hip prostheses [96] as comparatively smaller particles are found in hip prosthesis. MoM implants are found to reduce the potential for the induction of osteolysis [147] as MoM implants generate comparatively smaller particles. In addition, the small size of the wear particles may facilitate their dispersal via the lymphatic system to sites distant from the implant and it has been reported that cobalt-chrome particles can accumulate in the liver, spleen, lymph nodes, and bone marrow of patients [148,149]. Moreover, titanium nitride (TiN), chromium nitride (CrN), and chromium carbon nitride (CrCN) coatings applied on cobalt–chrome alloy (CoCr) substrate produces nano-size debris less than 40 nm. These wear particles showed reduced cytotoxic effect compared to the CoCr alloy debris cultured with U937 macrophages [75]. A dose-dependent reduction in bone resorption was achieved using human peripheral blood monocytes, cultured with osteoblast-like UMR 106 cells exposed to metal wear particles. This decrease in resorption was greater after exposure to CoCr and 316L-SS particles than to TiAlV and commercially pure Ti particles [135]. However, Sabokbar *et al*. [153] concluded that osteoclast formation is not significantly induced by particle characteristics (size, shape, and dose). Macrophage involvement in periprosthetic osteolysis also did not depend on particle phagocytosis. Zhang *et al*. [150] demonstrated that nano-sized ceramic particles were bioactive to cells, despite the significant secretion of inflammatory mediators from cells shown by nanoparticles of other materials (Figure 6). The aluminum nanoparticles significantly promoted the alkaline phosphatase (ALP) activity of the MG63 cells at a low concentration and did not show irritation to the macrophages. However, ALP activity of those treated with Ti microparticles was lower than that treated by ceramic nanoparticles. A larger volume of alumina particles (5 nm to 20 nm and a few >0.2 μm), from the hip joint simulator under micro-separation conditions, was required to activate the human peripheral blood mononuclear than the commercial alumina particle at 0.5 μm [151]. The critical particle size range to stimulate cell response was defined from 0.1 to 1 μm [136,151].

Debris from bone cements (CMW original, CMW1RO and Palacos R, CMW calcium phosphate, CMW copolymer bone cement) with sizes from 0.1 to 0.5 μm [134,140] were studied, and no statistical differences between the levels of bone resorption were induced by these cement types. Cements that contained pure CMW1 and CMW with calcium phosphate failed to induce the macrophages to express bone resorption activity, even at a high debris concentration (100:1 ratio). However, a major cytokine (TNF-α) was produced at the 100:1 ratio [134]. A similar study [140] demonstrated that bone cement particles are capable of inducing increase in TNF-α production *in vitro*, based on cement particle size, volume and cement particle type. Cement particles that contained radio-opaque additives were the most active. However, Baets *et al*. [152] demonstrated that metal debris occupied only 1.5% of total volume of wear debris retrieved from a cemented implant *in vivo* with 56.5% bony fragments and 42% cement fragments. The study prompts a rethink on the contribution of metal debris in bone resorption.

The inflammation and loosening of joint implants, incorporated with wear debris, result in the need for revision surgery. Deep infection results in severe complications and high economic burden. Demand for primary and revision joint replacements is expected to increase exponentially in the next two decades [155]. In addition, adjustable MoM bearings were found to be associated with a higher risk of periprosthetic joint infection when compared with CoC bearings [7]. Revision TJRs are associated with lower success rate, more complicated surgery and higher healthcare costs (by one third) [159] compared to initial TJRs surgery, which may induce additional damage to the surrounding tissues.

## 7. Discussion

Debris isolation needs to be carefully handled as the suitability of the isolation methods (alkaline, acidic, and enzymatic) is dependent on the type of prosthetic material. A minor change in particle morphology during isolation can change all the parameters of debris characterization. However, the enzymatic method was found to be the most user-friendly and effective in isolating the debris from conventional joint materials without any detrimental effects. Many researchers choose acidic/alkali methods because of the simplicity, reliability, speed, and material cost. Strong alkali can change the morphology of metal debris because metals are more active to alkali compared to UHMWPE and ceramic debris, which are mostly inert. Centrifugation, dilution, ultrasonication, heating, and filtration are common steps in the particle isolation process chain (Figure 2). Histological analysis is also effective in characterizing and quantifying debris without performing an isolation method.

Particle imaging, whether in histological or isolated form, is a major step in debris characterization. Advanced 3D imaging is preferred over the old 2D characterization due to the visual aspects and the reliability in particle quantification of 3D imaging. Several debris quantification methods have been developed in the last decade, where SEM, IR, and AFM were used as input. The shape and size of debris are mostly defined, based on their ECD and AR. The frequency distribution of particle sizes and the relation between the sizes and volume or the sizes as well as number of particles were intensively studied.

The morphology of particles is significantly dependent on the type of joint (knee/hip), bearing (fixed/mobile), material (UHMWPE, metals, and ceramics), bearing couple (MoM, MoC, MoP, CoC, and CoP), experimental environment (*in vitro*/*in vivo*), and other parameters (loadings and lubricants). Knee prostheses produce larger particles than hip prostheses, possibly because of the variation in the loads, contact area, direction of the movements and difference in wear mechanisms between these two joints. Debris retrieved from a hip joint simulator was larger in size and different in shape compared with the particles isolated from periprosthetic tissues [99,104]. The discrepancy of the wear debris morphology obtained from different sources raises the question of the validity of the joint simulator. In addition protein level and the viscosity of the lubricant in the joint simulator may affect debris morphology as the study exhibited smaller and thinner particles in serum than in water as lubricant [122,124]. In this circumstance, the morphology of wear debris plays an important role in validating a newly designed joint simulator. MoM and CoC prostheses produced relatively smaller debris than that in polyethylene-oriented material combinations, possibly because of the softness of polyethylene compared with the counterpart material. No considerable difference in debris size was found between the mobile and fixed bearings from the knee simulator and the knee prosthesis. The particle became spherical or mostly round in shape when the particles are smaller and is subjected to third body abrasive wear. A huge variation in size and shape was found among the isolated particles in the same cohort from the periprosthetic tissues as shown in Figures 3 and 4. The nanometer-sized particles were relatively round and spherical compared with the micro-sized particles, which were elongated, fibril, and flake-shaped.

The evidence reveals that the most important cellular target of wear debris is the macrophage. Macrophages are located in the interfacial membrane between the joint and the bone, where wear particles are actively ingested [160]. Periprosthetic osteolysis was reinforced by the inflammatory factors and the systemic levels (such as hormones, growth factors, cytokines, and loading patterns), where the key role was attributed to the macrophages [161]. The particles around the joint prosthesis inhibited the activities of the osteoblasts and activated osteoclasts, which induced matrix deposition and mineralization followed by bone resorption. The phagocytes produced inflammatory mediators/secreted factors such as TNF-α, RANKL, IL-6, PGE_2_, and IL-1b, which were implicated in osteoclast activation and bone resorption.

**Figure 8.**
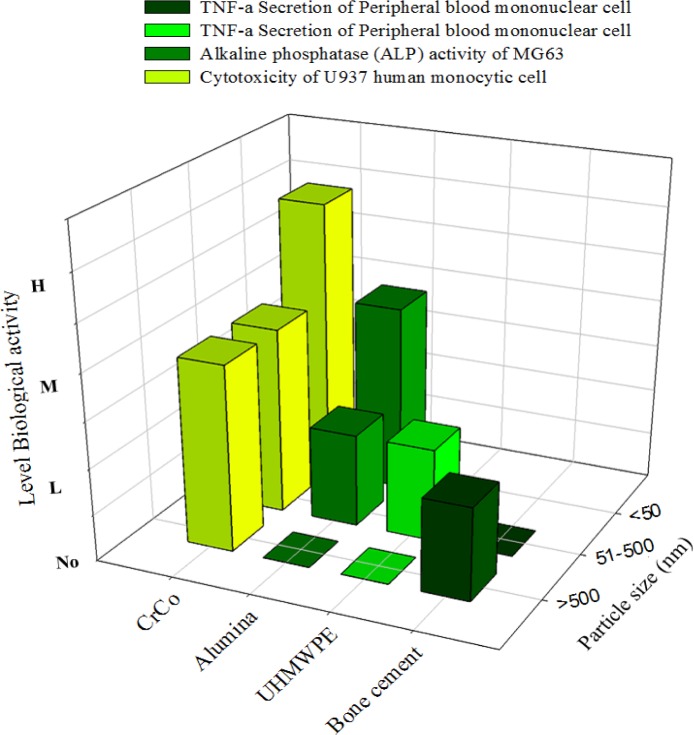
Size dependent biological response of wear particles (based on Table 4).

Cell proliferation, differentiation, and prostanoid are affected by the size, shape, and chemical composition of the particles. The nanoparticles disintegrate within the cells more rapidly and induce more damage. The incubation of metal nanoparticles with cells releases ions in the culture medium, which results in mineralization (Figure 7) to reinforce bone resorption. Polyethylene particles with sizes ranging from nano to micro do not affect cell viability, but submicron size polyethylene particles strongly influence the secretion of cytokines. The debris from bone cements at a high concentration can influence the production of TNF-α. A few studies defined that the critical particle size range that stimulates cell response is between 0.1 μm and 1 μm. However, the stimulation of cell and bone resorption is significantly dependent on dose and sized of particles (Figure 8). The phagocytosis of the particles is size-dependent [145], and higher dose of particles can be ingested by the macrophage when the particles are relatively smaller. Higher doses of particles of any material have adverse effect on cell viability and proliferation with high secretion of different types of mediators that activate osteoclasts, thereby resulting in mineralization followed by osteolysis and implant failure.

## 8. Limitation

Debris formation varies based on the material, bearing, lubricant, and other common parameters such as joint type, force, and contact area. Newly-developed advanced materials, such as the carbon-carbon composite [110,154], CFR PEEK [162], and PCU [163] were proposed for use in hip or knee prostheses. Therefore, evaluating these advanced materials by characterizing debris with their corresponding biological response is necessary. Noble engineering techniques were also employed on materials to improve the surface in terms of wear rate, biocompatibility, and affordability. A patterned surface (such as dimple, ripple, square grid, and spider net) [81–83] and coating (diamond-like carbon, micronite, and diamond) [71–74,77–80] were studied *in vitro* more than decade ago. However, only a few articles [71,75,164] that characterize wear debris retrieved from the patterned and coated surfaces employed in hip and knee prosthesis were found. *In vitro* macrophage responses to nano-diamond particles [165] were investigated and were found to significantly reduce the gene expression of TNF-α. Comparative studies on the morphological and biological characterizations of wear debris from patterned and non-pattered or coated and non-coated surfaces are warranted.

## 9. Conclusions

A review of investigations of different materials with different wear mechanism used in hip and knee arthroplasty was conducted to correlate the findings relevant to debris morphology and their disintegration into periprosthetic tissue. The findings from the overview are summarized below.

- An appropriate process for debris characterization involved the correct protocol for debris isolation, followed by advanced imaging procedure, quantification, and utilization of the correct statistical data representation. An inappropriate isolation method may have detrimental effects on the size, shape, and number of particles.
- The particles generated were neither uniform in size and shape nor homogeneous in distribution, both *in vivo* and *in vitro*. The debris size, ranging from nanometers to micrometers, varied in shape and volume depending on the type of joint (knee/hip or mobile/fixed), bulk material and their combination, wear mechanism, and experiment conditions (load, speed, and lubrication). The most common debris shapes were spherical, cylindrical, fibril, and flake.
- Debris retrieved from polyethylene, metal and ceramics implants showed higher inflammatory response to living cells when they were smaller in size. In addition, phagocytosis of particles is found to be debris-sized-dependent. Therefore, the nano-sized wear particles retrieved from any prosthesis material are expected to be highly capable of stimulating cells at a given high volumetric dose. The size-dependent response rate weakens with lower doses.

## Figures and Tables

**Table 1.** Protocols for particle isolation.

Materials	Digestion Methods
UHMWPE	Alkaline [Sodium Hydroxide ( (NaOH)] [87]
Ceramics	Acidic [Nitric acid (HNO_3_)] [88]
UHMWPE	Alkaline [Sodium Hydroxide (NaOH)] [90] Alkaline [Sodium Hydroxide (NaOH)] [89]
UHMWPE	Acidic [Hydrochloric acid (HCl)] Enzymatic [Proteinase K] Enzymatic [Papain+ Proteinase K] [94]
Metal	Alkaline [Potassium/Sodium Hydroxide (KOH)/(NaOH)]
UHMWPE	Alkaline [Sodium Hydroxide (NaOH)] [97]
UHMWPE	Alkaline [Potassium Hydroxide (KOH)] [96] Alkaline [Potassium/Sodium Hydroxide (KOH)/(NaOH)] [91]
UHMWPE	Acidic [Nitric acid (HNO3)/Hydrochloric acid (HCl)] Enzymatic [Proteinase K]
Metal	Enzymatic [Papain + Proteinase K +yeast lytic enzyme + Zymolyase] [93]
UHMWPE	Acidic [Nitric acid (HNO3)] [92] Alkaline [Potassium/Sodium Hydroxide (KOH)/(NaOH)] [100]
UHMWPE	Acidic [Nitric acid (HNO3)] Enzymatic [Proteinase K + Liberase Blendzyme 3]
UHMWPE	Alkaline [ Sodium Hydroxide (NaOH)] [99]
UHMWPE	Enzymatic [Papain] [105]
Metal	Enzymatic [Papain + Proteinase K] [106]

**Table 2.** Characterization of Polyethylene wear debris from different types of bearing.

Materials	Bearing Type	Sources	Shape	Size	Instruments
UHMWPE [96] (crosslinked)	knee joint	simulator	spherical and flakes	0.1–1 μm	FEGSEM
	hip joint			<0.1 μm	

UHMWPE [104]	hip joint	periprsosthetic tissues	irregular	75% < 0.5 μm; 90% < 1 μm	TEM
	knee joint			43% < 0.5 μm; 72% < 1 μm	

UHMWPE [99]	hip joint	periprsosthetic tissues	AR,1.626 ±0.015	ECD, 0.694 ± 0.005 μm	SEM
	knee joint		AR, 1.935± 0.015	ECD, 1.190 ± 0.009 μm	

Polyethylene [114]	mobile bearings	knee joint simulator	AR, 1.853 ± 0.877; roundness, 0.528 ± 0.152	0.074–1.319 μm, ECD = 0.265 ± 0.131 μm	FE-SEM
	fixed bearings		AR,1.926 ± 0.712; roundness, 0.494 ± 0.169	0.013–1.120 μm, ECD = 0.270 ± 0.148 μm	

UHMWPE [115]	mobile bearing TKAs	synovial fluids of patients	AR, 1.94 ± 0.13and roundedness,1.92 ± 0.18	ECD,0.81 ± 0.12 μm	SEM, Image analyzer
	posterior stabilized TKAs		AR, 2.30 ± 0.22 and roundedness, 2.52 ± 0.36	ECD, 0.78 ± 0.08 μm	

UHMWPE (with CoCrMo alloy) [34]	hip joint	implanted	spherical, sub-spherical, plate structure	0.5–5 μm with Avg. dia. 1.33 μm	LPSA, SEM, TEM
		simulator	strip, block, plate, and spherical	4–20 μm with Avg. dia. 7.54 μm	

UHMWPE [127]	alumina medial pivot	total knee prosthesis	AR, 1.52 ± 0.05 and roundness, 1.34 ± 0.05	ECD, 0.78 ± 0.4 μm	SEM, image analyzer
	CrCo alloy medial Pivot		AR, 1.88 ± 0.11 and roundness, 1.75 ± 0.12	ECD, 0.66 ± 0.06 μm	

UHMWPE [118]	multidirectional pin on plate rig	crosslinked	spherical	<100 nm	FEGSEM
		non-crosslinked		0.1–1 μm	

**Table 3.** Characterization of wear debris of different materials.

Materials	Type	Source	Shape	Size	Instruments
Carbon/carbon composite [110]	needled carbon cloth	hip joint simulator	broken and fragment fiber, cylindrical, slice and spherical pyrolytic	24.8% > 5 μm, 67.7% is 5–30 μm, 7.5% < 30 μm	LPSA, SEM
	carbon felt			36.4% > 5 μm, 59.8% is 5–30 μm; 3.8% < 30 μm	

UHMWPE (with Standard size CoCr) [126]	mobile bearings	knee joint simulator	elongated, fibril like and spherical	0.2–0.8 μm	AFM, SEM

CoCrMo alloy [93]	–	hip joint simulator	rounded and irregular	<50 nm	SEM, TEM

UHMWPE [119]	revisions surgery of THRs	periprosthetic tissues	cylindrical, slice and spherical	0.1–10 μm and <10 μm	SEM, IR, EDX/EDS

UHMWPE(on Al2O3, 316L stainless steel, CoCrMo alloy, Ti6Al4V head) [120]	mobile bearings	hip joint simulator	round, flake like, stick, twig debris	Frequently occurs within range of 1–30 μm, but overall size range is 0.1–320 μm	SEM, EDS

UHMWPE [92]	revisions surgery of THRs	periprosthetic tissues	elongation, 1.29± 0.13, 1.35 ± 0.29 and circularity, 0.97 ± 0.07, 0.93 ± 0.09	ECD, 18.5 ± 5.29 nm and 21.2 ± 8.01 nm	FEGSEM, EDS, IR

CoCrMo (Metal on Metal) [106]	revisions surgery of THRs	periprosthetic tissues	needle shaped	40–120 nm	SEM, HR-TEM, EDS, XPS
			globular	≤90 nm	

UHMWPE [121]	revisions surgery of THRs	periprosthetic tissues	rounded, fibril and flake	<35%, 30 nm and 0.1–0.99 μm, rests are > 1 μm	FEGSEM, EDS

UHMWPE [105]	revisions surgery of THRs	periprosthetic tissues	rounded, flattened and flakes or fibrils	87.9% < 1 μm	TEM, SEM

UHMWPE [101]	hip joint	periprosthetic tissues	rounded, beads, fibrils, flakes	ECD range is from 0.48 to 0.95 μm	SEM, Micro-Raman spectrometry

CoCrMo alloys [98]	high carbon	hip joint simulator	round, oval and needle shaped	Length, 48 ± 28 nm	TEM, EDX
	low carbon			Length, 57 ± 27 nm	
	cast			Length, 53 ± 26 nm	
Alumina [103]	hip joint	periprosthetic tissues	polygonal	5–90 nm and 0.05–2 μm	TEM, SEM,EDX, LCM
UHMWPE [112]	hip joint	periprsosthetic tissues of thrs	fibril, platelet	Most of particls, 0.1–0.5 μm and very few >10 μm	SEM
TiN, CrN, CrCN coating on CrCo alloy [75]	hip joint	multidirectional pin-on-plate tests	round	<40 nm	SEM

**Table 4.** Biological response of wear debris on human cells.

Materials	Size	Cell type	Bioactivity			Sources
Alumina [150]	40–50 nm, purity 99.5%	human osteoblasts (MG-63)	**ALP**			Commercial powder
			active at low concentration			
Zirconia(IV)	<50 nm		active at high concentration			
Silicon nitride	<50 nm, Purity 98%		active at high concentration			
Titanium	<20 μm, Purity 93%		Not so active			

CoCr alloy [139]	29.5 ± 6.3 nm	Human dermal fibroblasts	**Genotoxicity**	**Cytotoxicity**		Flat pin-on-plate Tribometer

		more DNA damage	low response			
	2.904 ± 1.064 μm	less DNA damage	low response			

CoCr alloy [144]	2–5 μm	Human dermal fibroblasts	**Genotoxicity**			Commercial alloy powder
			Significant DNA damage			

FeAlCr alloys [145]	(7.5, 3.7) ± 0.4 μm	human osteoblast (SAOS-2)	**Viability**	**Proliferation**		Commercial alloy powder

			good at 1st 24 h then decreased	good at 1st 24 h then decreased		
PM 2000 (Fe base alloy)	18.4 ± 0.4 μm		Good	Good		
Ti6Al4V alloy	Avg. 150 μm		good at 1st 24 h	good at 1st 24 h		

–	–	U937 human monocytic cell	response to Caspase-3	response to Caspase-8		Laboratory
Co^2+^ ions [141]			significant effect after 24 h of cubation	No effect		
Cr^3+^ ions			significant effect after 4 h of cubation but 50% of Co^2+^ ions	increased after 2 h cubation, gets max. after 8 h		

Clinical CoCr alloy [138]	29.5 ± 6.3 nm	U937 (human) histiocytic cell	**Viability**			Flat pin-on-plate tribometer and commercial powders
			43% reduced by day 1 and 97% by days 3 at 50 μm^3^/cell			
Clinical alumina	5–20 nm		18% reduced from day 4 at 50 μm^3^/cell			
Commercial CoCr alloy	9.87 ± 5.67 μm	U937 (human) histiocytic cell	27% reduced by 4 days at 50 μm^3^/cell and no response at low concentration			
Commercial alumina	0.503 ± 0.19 μm		no response at any concentration			

Alumina [151]	–	peripheral blood mononuclear cell (PBMC)	**TNF-α Secretion**			micro-separated particles
	5–20 nm		significant level when stimulated with higher volume of particles but showed low respond to microseparation wear particles			
	and 0.5 μm					commercial powder

–	0.1–1 μm, 0.1–10 μm; 1–10 μm, >10 μm	peripheral blood mononuclear cell (PBMC)	**TNF-α Secretion**			uni-directional pin on plate
CMW original [140]			failed to stimulate at any size			
CMW1 RO			greater response at 0.1–1 μm			
Palacos R			less active than CMW1RO			

–	–	–	**Cytotoxicity**			–
CoCr alloy [154]	53 nm	–	more than P25-CVD			
P25-CVD	24.2 ± 13 nm	U937 human monocytic cell	low response			

UHMWPE [136]	0.21 μm	peripheral blood mononuclear cell (PBMC)	**Viability**		**TNF-α Secretion**	–

			unaffected by any size of the particle		significant	commercial powder
	0.49 μm				significant	
	4.3 μm				no secretion	
	7.2 μm				no secretion	
	88 μm				no secretion	

–	68% to 83% of particles, 0.1–0.5 μm	U937 human monocytic cell	**Bone resorption**	**TNF-α Secretion**		uni-directional pin on plate

CMW1original [134]			significant	secretion increases with increasing particle feed in case of all type of bone cement debris		
CMW1RO			significant			
CMW copolymer 1			significant			
CMW copolymer 2			significant			
Palacos R			significant			
CMW CaPO4 20%			not significant			
CMWCaPO4 30%			not significant			
